# Endoscopic treatment of ganglioneuroma of the colon associated with a lipoma: a case report

**DOI:** 10.1186/1752-1947-6-304

**Published:** 2012-09-14

**Authors:** Enrico Fiori, Chiara Pozzessere, Antonietta Lamazza, Giovanni Leone, Francesco Borrini, Alberto Schillaci, Pietro Mingazzini

**Affiliations:** 1 Department of Surgery “Pietro Valdoni”, University of Rome “Sapienza”, Viale del Policlinico 155, 00161, Rome, Italy

## Abstract

**Introduction:**

Ganglioneuromas are rare benign peripheral neuroblastic tumors characterized by hyperplasia of ganglion cells, nerve fibers, and supporting cells. They are not usually localized in the colon.

**Case presentation:**

A 61-year-old Caucasian man was admitted to our department for colon cancer screening. A colonoscopy revealed a lipoma of 5cm in diameter, two micropolyps of less than 1cm, and one sessile polyp of 0.6cm in diameter. The polyps were removed with hot biopsy forceps. A histological examination revealed two hyperplastic polyps and one ganglioneuroma polyp. A follow-up colonoscopy showed no signs of recurrence after 16 months.

**Conclusions:**

Although a few cases of lipomas associated with ganglioneuromatous syndrome have been reported, the association of an intestinal lipoma with an isolated ganglioneuroma polyp has not been described. The implications of this association are unknown.

## Introduction

Ganglioneuromas (GNs) are slow-growing and well-differentiated neuroectodermal neoplasias. They are derived from developing neuronal cells of the sympathetic nervous system and occur mostly in children. GNs can be detected in different anatomical locations but are rarely found in the colon. Intestinal GNs have been found in patients with several systemic syndromes such as multiple endocrine neoplasia type IIB (MEN IIB), neurofibromatosis type 1 (NF1) (also known as von Recklinghausen’s disease), juvenile polyposis, polyposis coli, tuberous sclerosis, and Cowden’s disease [1]. We report the case of a patient with synchronous GN and lipoma. Cutaneous and intestinal lipomas have been associated with GN polyposis in a few cases, but the implications of this association are unknown.

## Case presentation

**Figure 1:**
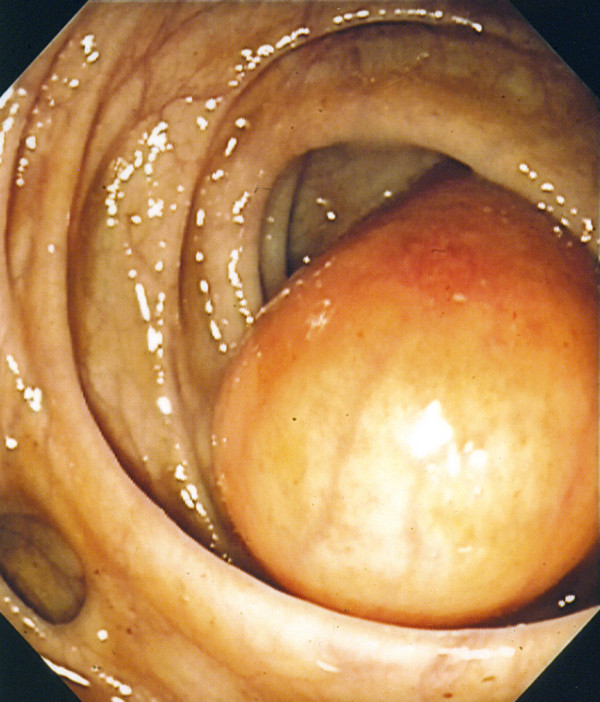
** Endoscopic image of lipoma and diverticula.** A yellowish spherical submucosal lesion is observed in the descending colon. Diverticula are evident

**Figure 2:**
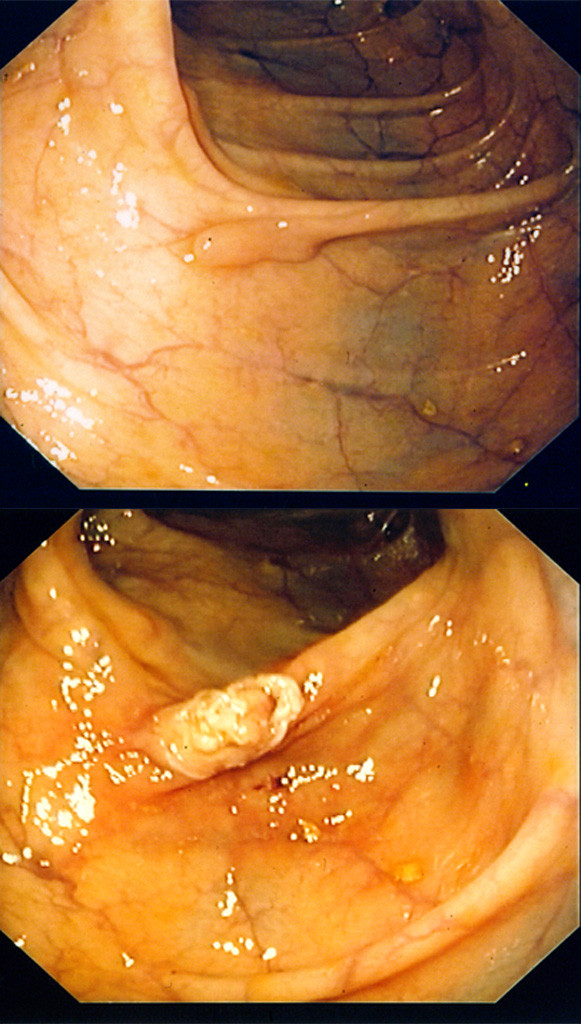
** Endoscopic image of the polyp. (a)** A sessile polyp with a diameter of 0.6cm is observed in the descending colon. **(b)** The polyp is seen here after removal with hot biopsy forceps

**Figure 3:**
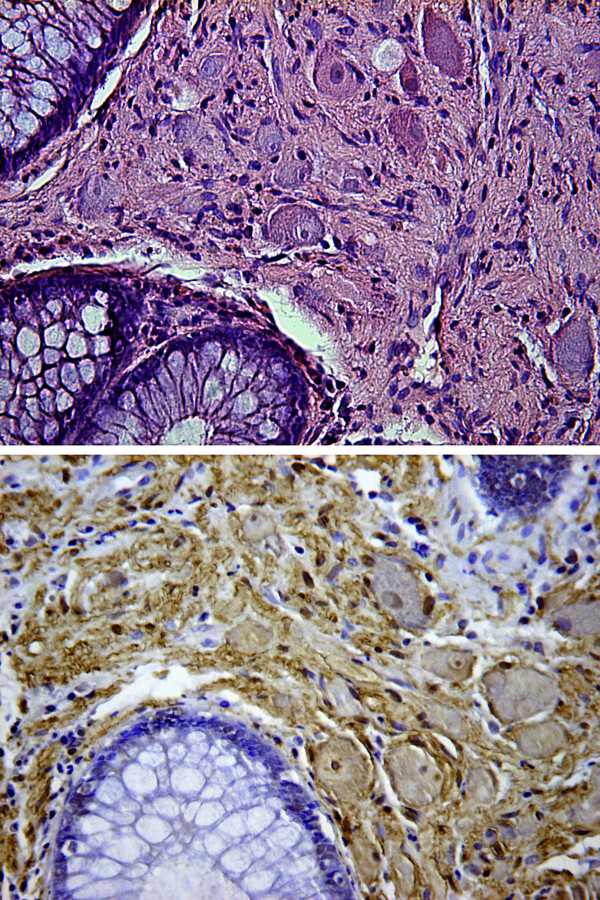
** Histological examination of the ganglioneuroma polyp. (a)** Proliferation of spindle and ganglion cells in the lamina propria displaced the glands (stain: hematoxylin and eosin; magnification: ×200). **(b)** The spindle cells demonstrated immunoreactivity to anti-S100 protein (magnification: ×200)

A 61-year-old Caucasian man underwent colonoscopy for colon cancer screening. No abdominal or intestinal symptoms were reported, and his family history, medical history, and laboratory test results were negative for bowel cancer. A yellowish spherical submucosal lesion that was 5cm in diameter and that arose from the descending colon was detected. It was subsequently identified as a lipoma (Figure 1). Several micropolyps (of less than 1cm) with a normal overlying mucosa were revealed; two of these were found in the hepatic flexure, and one sessile polyp with a diameter of 0.6cm was found in the descending colon (Figure 2). All were removed with hot biopsy forceps. Multiple diverticula were also found. The polyps from the hepatic flexure were microscopically identified as hyperplastic polyps. The polyp from the descending colon showed crypt rarefaction and distortion, an expanded lamina composed of S100-positive spindle cells, and numerous isolated ganglion cells (Figures 3). Histological diagnosis was an isolated polypoid GN of the large bowel. Diagnosis of the lipoma was performed by endoscopic examination alone. After histological diagnosis of the GN, our patient was examined for other systemic and familial diseases. The results of familial anamnesis and clinical examination were negative for other pathological associations. A follow-up colonoscopy performed after 16 months revealed no signs of recurrence.

## Discussion

Intestinal GNs are hamartomatous polyps characterized by hyperplasia of ganglion cells, nerve fibers, and supporting cells of the enteric nervous system [2]. Shekitka and Sobin [3] categorized GNs of the intestinal tract into three groups: polypoid GN, ganglioneuromatous polyposis, and diffuse ganglioneuromatosis. Polypoid GNs can be solitary or few in number. They are often small and may be sessile or pedunculated. They are endoscopically indistinguishable from hyperplastic or adenomatous polyps. Occasionally, they occur in patients with Cowden’s disease, tuberous sclerosis, polyposis coli, or juvenile polyposis [4]. More than 20 sessile or pedunculated polyps develop in patients with ganglioneuromatous polyposis. These polyps may be an isolated finding but more commonly are identified as a component of one of the following syndromes: MEN IIB syndrome, NF, Cowden’s disease, or Ruvalcaba-Myhre-Smith syndrome. The presence of multiple cutaneous lipomas or skin tags or both has also been reported [2,3]. Diffuse ganglioneuromatosis disseminates to the entire colon but does not involve the ileum. Lesions can reach 1 to 17cm in diameter and may comprise multiple colonic polyps and nodular intramural or transmural proliferation of neural elements that involve the enteric plexuses. These lesions are poorly demarcated and can distort the architecture of the surrounding tissue [5]. Although they may be an isolated finding, they are often observed as a component of MEN IIB7 or NF 1 [6]. Hegstrom and Kircher [7] detailed an autopsy case of diffuse gastrointestinal ganglioneuromatosis-lipomatosis. Treatment of GNs depends on the clinical history; polypectomy is curative for polypoid subgroups, but colectomy may be necessary for polyposis or diffuse forms [8]. In this report, a solitary polypoid GN was incidentally detected during endoscopy. In most cases, a solitary polypoid GN is associated with no characteristic symptoms. However, depending on its size and anatomical location, it may cause abdominal pain, constipation, obstruction, or bleeding [4,9,10]. Furthermore, GN has no typical endoscopic appearance. In this case, it was confined to the mucosa and was overlaid with normal colonic mucosa. Microscopic examination and immunohistochemistry enabled the definitive diagnosis. Stains for S100 protein and neuron-specific enolase confirm the presence of spindle cells and ganglion cells, respectively. There are no specific treatment recommendations for GNs, but they may be treated according to their size, location, and complications such as bleeding or obstruction or both. In this case, polypectomy was performed with biopsy forceps, and the six-month follow-up showed no signs of recurrence. Because of the absence of associated pathologies or symptoms, clinical management comprised a follow-up colonoscopy 18 months later. Some authors have reported the coexistence of colon cancer with GN polyposis in patients with diffuse ganglioneuromatosis [11-13]. However, this association is controversial and, to some investigators, unacceptable [14]. In a recent study of colon adenocarcinoma associated with diffuse ganglioneuromatosis, a glial cell line-derived neurotrophic factor (GDNF) and its receptor components were identified. GDNF family receptor alpha 1 (GFR-alpha 1) and the receptor tyrosine kinase (RET) are involved in the pathophysiology of both the enteric nervous system and adenocarcinoma cells, suggesting their involvement in the pathology of polypoid GN [15]. The association between colon cancer and polypoid GN has not been studied before. The variant discussed in the present report may be cured by endoscopic resection [9].

## Conclusions

Because of the link between polypoid GN and other systemic diseases, patients should be carefully screened for both associated syndromes and malignancies in the colon and other locations, including the thyroid, breast, and uterus [8]. However, some authors feel that this screening is unnecessary because of the benign nature of polypoid GN [16]. In this study, we found a lipoma during colonoscopy. Cutaneous and intestinal lipomas have been detected in a few cases of polyposis [1,3,7] and diffuse GN subgroups. However, they have never been described in the solitary subgroup. Furthermore, the nature and significance of this association are unclear. We presume that there was no relationship between polypoid GN and lipoma in this particular case and that their occurrences were likely coincidental.

## Consent

Written informed consent was obtained from the patient for the publication of this case report and accompanying images. A copy of the written consent is available for review by the Editor-in-Chief of this journal.

## Abbreviations

GDNF: glial cell line-derived neurotrophic factor; GN: ganglioneuroma; MEN IIB: multiple endocrine neoplasia type IIB; NF 1: neurofibromatosis type 1.

## Competing interests

The authors declare that they have no competing interests.

## Authors’ contributions

EF performed endoscopy, made a major contribution to writing the manuscript, and gave final approval of the version to be published. CP performed the literature review and acquired data. AL performed the literature review and the critical review. GL prepared the manuscript and performed the critical review. FB and PM described the histology. AS gave final approval of the version to be published. All authors read and approved the final manuscript.
